# Erythropoietin Effect on Complement Activation in Chronic Kidney Disease

**DOI:** 10.3390/biomedicines12081746

**Published:** 2024-08-02

**Authors:** Virginia Athanasiadou, Kleio Ampelakiotou, Eirini Grigoriou, Katherina Psarra, Alexandra Tsirogianni, Serena Valsami, Theodoros Pittaras, Eirini Grapsa, Maria G. Detsika

**Affiliations:** 1 Department of Nephrology, School of Medicine, Aretaieion University Hospital, National and Kapodistrian University of Athens, 11528 Athens, Greece; virg.athanasiadou@gmail.com (V.A.); egrapsa@med.uoa.gr (E.G.); 2 Department of Immunology and Histocompatibility, ‘Evangelismos’ General Hospital, 10676 Athens, Greece; k.ampelakiotou@gmail.com (K.A.); egrigore@otenet.gr (E.G.); kpsarra@outlook.com (K.P.); alextsir@gmail.com (A.T.); 3 Hematology Laboratory-Blood Bank, Aretaieion Hospital, National and Kapodistrian University of Athens, 11528 Athens, Greece; serenavalsami@yahoo.com (S.V.); teopittaras@yahoo.gr (T.P.); 4 1st Department of Critical Care Medicine and Pulmonary Services, GP Livanos and M. Simou Laboratories, Evangelismos Hospital, National and Kapodistrian University of Athens, 10675 Athens, Greece

**Keywords:** complement, chronic kidney disease, lymphocytes, erythropoietin

## Abstract

The complement system is an important part of innate immunity. Despite its known protective role, the complement system may contribute to increased inflammation and tissue injury in cases where its balanced activation is disrupted. The kidneys have been shown to be largely affected by complement dysregulation. The aim of the present study was to investigate the effect of erythropoietin administration, on the complement system, in chronic kidney disease patients. The study involved 20 patients with CKD who received erythropoietin and measurements of levels of complement factors C3a and C5a and complement regulatory proteins (CregPs) CD55, CD46, and CD59. An increase in serum C3a and C5a levels was observed in response to EPO therapy. The increase in C3a was statistically significant (*p* < 0.05) and concurrent with a statistically significant decrease in CD55 in CD4^+^ T cells (*p* < 0.05) and B cells (*p* < 0.05) and CD59 levels in CD4^+^ and CD8^+^ T cells (*p* < 0.05) at completion of EPO therapy compared with healthy controls. The above observations demonstrate that EPO induces complement activation in patients undergoing EPO therapy with a simultaneous restriction of CRegPs expression, thus possibly allowing the uncontrolled complement activation, which may contribute to tissue injury and disease progression.

## 1. Introduction

Although a key function of the complement system is the recognition and elimination of pathogens via direct killing and/or stimulation of phagocytosis, activation of this system also results in tissue injury and disease [1]. It is now well established that excessive activation or insufficient control of complement activation on host cells can cause an immune imbalance that may fuel a vicious cycle between complement, inflammatory cells, and tissue damage, thereby exacerbating clinical manifestations and outcomes of disease [2].

There are three pathways of complement activation: the classical pathway, the alternative pathway, and the lectin pathway. All three pathways consist of a series of reactions in which one molecule cleaves and activates the next, leading to the terminal step involving cell lysis [3]. Although initially different, the three pathways converge at the C3 step and follow the same steps until the formation of the terminal complement component, known as the membrane attack complex (MAC, C5b-9). The complement system is known for its protective role against circulating pathogens, such as bacteria and viruses; however, in an imbalanced cascade, its activation may lead to increased inflammation, tissue damage, and disease progression. This is why the activation of the complement system is subjected to control by a group of proteins known as complement regulatory proteins (CRegPs), which halt complement overactivation and maintain a balanced cascade [4,5].

In humans, CRegPs are both membrane-bound and fluid-phase. There are four membrane-bound CRegPs: decay accelerating factor (DAF, CD55), membrane co-factor protein (MCP, CD46), complement receptor 1 (CR1), and CD59. CRegPs are expressed in all cell types, including blood mononuclear cells such as monocytes, T cells, B cells, erythrocytes, and granulocytes [6,7].

The kidneys are known to be widely affected by complement cascade imbalances, and various kidney diseases are mediated directly by imbalanced complement activation, ultimately leading to chronic kidney disease (CKD) [8,9]. One of the hallmark complications of CKD is anemia, primarily due to decreased production of erythropoietin (EPO) by the damaged kidneys. Thus, EPO therapy is commonly used to treat anemia in CKD patients. EPO therapy aims to alleviate anemia by stimulating erythropoiesis, thereby improving tissue oxygenation and mitigating renal hypoxia. By restoring tissue oxygen delivery, EPO treatment may attenuate renal injury and slow the progression of CKD. Moreover, emerging evidence suggests that EPO possesses direct renoprotective properties independent of its hematopoietic effects. Preclinical studies have demonstrated that EPO receptors are expressed not only on erythroid progenitor cells but also on various renal cell types, including tubular epithelial cells, endothelial cells, and mesangial cells. The activation of these receptors by exogenous EPO can trigger intracellular signaling pathways involved in cell survival, proliferation, and anti-apoptotic mechanisms. Experimental models of CKD have shown that EPO administration attenuates renal inflammation, oxidative stress, and apoptosis while promoting tubular cell regeneration and angiogenesis. These pleiotropic effects collectively contribute to renal tissue preservation and functional recovery, thereby potentially slowing the progression of CKD. Furthermore, EPO treatment may mitigate other CKD-related complications beyond anemia, such as cardiovascular disease and proteinuria. Anemia is a significant risk factor for adverse cardiovascular events in CKD patients, and EPO therapy has been associated with improvements in left ventricular function, exercise tolerance, and overall cardiovascular outcomes. Additionally, studies have suggested that EPO therapy may reduce proteinuria, a marker of renal injury and progression, through mechanisms involving the modulation of podocyte function and preservation of glomerular integrity. EPO therapy exerts multifaceted effects on CKD, including the correction of anemia, tissue protection, anti-inflammatory actions, and mitigation of ischemia-reperfusion injury [10,11].

The effect of erythropoietin therapy on complement in CKD patients remains unknown. Previous reports have only described changes in CRegPs expression levels in the erythrocytes of CKD patients after EPO therapy with an upregulation of CD55, CD59, and CR1 in response to EPO treatment [12,13]. However, the impact of EPO therapy on complement levels in association with changes in CRegPs has not been investigated before. The current study addressed this question by assessing complement factors and CregPs levels in CKD patients receiving EPO therapy. 

## 2. Materials and Methods

### 2.1. Patients

This study was reviewed by the Institutional Ethics Committee, and the need for approval was granted (board name: Scientific and Ethics Committee of Aretaieion Hospital, National and Kapodistrian University of Athens, approval number: 193/25-02-2020, approval date: 25 February 2020, study title: The effect of erythropoietin on the immune profile of patients with chronic kidney disease). All procedures were carried out in compliance with the Helsinki Declaration. Informed written consent was obtained from all patients or the patients’ next of kin. A total of 20 patients were included, of whom 10 were male and 8 were female, and 20 healthy controls, of whom 10 were male and 10 were female. The mean age of patients was 73.30 ± 14.21 and the mean age of the healthy controls was 60.35 ± 14.88 ** (*p* < 0.01 compared to the patient’s group). All patients had CKD—stages 3B to 5—of various pathologies, as shown in Table 1. These included hypertensive nephropathy, diabetic nephropathy, polycystic kidney disease, membranous nephropathy, and IgA nephropathy. The various pathologies equally affected the female and male patients enrolled in the study, with the exception of polycystic kidney disease, which was the main cause of CKD in two male patients enrolled in the study. Hypertensive nephropathy was the main cause of CKD in the patient sample analyzed in accordance with the official data from the EMENO study, which stated that the prevalence of arterial hypertension in Greece was approximately 40% of the adult population, while diabetes mellitus was around 12% [14,15]. All 20 patients were treated with angiotensin-converting enzyme inhibitors (ACEi) or angiotensin receptor blockers (ARBs) to treat hypertension and/or albuminuria. Furthermore, three patients with hypertension also received β-blockers, while the remaining hypertensive patients and two patients with polycystic kidney disease received triple therapy with ACEi, calcium channel blockers (CCBs), and diuretics. Comorbidities included coronary heart disease (two hypertensive patients received non-vitamin-K-antagonist oral anticoagulants (NOACs), chronic obstructive pulmonary disease (one patient with polycystic kidneys and one with diabetes received beta-2 agonist inhalers), and depression (a membranous nephropathy patient and a diabetic patient received escitalopram and venlafaxine, respectively). The selection criteria for the healthy individuals were no comorbidities and no treatment received. 

Prior to the initiation of therapy, blood samples were collected, and complete blood counts, including hemoglobin (Hb) concentration and hematocrit (Ht), red cell indices, platelet count, white blood cell count, and differentials, along with serum ferritin levels, vitamin B12, and folate levels, were determined. According to international guidelines for CKD patients, ferritin levels should be ≥500 ng/mL, vitamin B12 levels should be ≥1000 pg/mL, folate levels should be ≥20 ng/mL, and Hb concentration should be between 9.0–10.0 g/dL in order to initiate therapy with recombinant erythropoietin [16]. No patient received vitamin B12, folate, or ferrum supplements, as all patient levels for these parameters remained within the normal ranges throughout the study.

Patients received recombinant human erythropoietin alfa. The recommended dose for the specific erythropoietin alpha by the SPC is 50–100 units/kg subcutaneously three times a week. All patients recruited in the study were consistently administered a dose of 75 units/kg three times per week subcutaneously. Blood samples were collected in Aretaieio Hospital and transported immediately to the hospital laboratory for analysis on a monthly basis until the patient reached a Hb goal level of 11 g/dL for women and 11.5 g/dL for men. Patients who reported a headache or a sudden increase in blood pressure were recalled earlier for a follow-up. Demographics, clinical data, and Hb/Ht values per blood draw were recorded. During the course of EPO therapy, one patient passed away, and one patient could not reach our clinic, for the required therapy and tests, due to COVID-19 restrictions. These two patients were omitted from the study. 

### 2.2. Flow Cytometric Analysis for CD55, CD46, and CD59 Protein Expression

The study focused on cellular CRegPs, CD55, CD46, and CD59, as these are readily expressed in blood cell populations [6]. Whole blood samples were obtained from patients prior to the initiation of therapy and at completion of therapy. 900 μλ of whole blood sample was added to 100 μλ of DMSO and samples were stored at −80 °C until further use. Blood samples were thawed in a 30 °C water bath and diluted with pre-warmed PBS 1:5. Diluted blood samples were centrifuged at 800 rpm for 5 min and resuspended in fresh, prewarmed PBS (5 mL). Typically, 1 mL of diluted blood sample was added to standard volumes of antibodies (Supplemental Table S1) and incubated in the dark for 15 min. Upon completion of incubation, FACS lysing solution (BD Pharmigen, BD Biosciences, Franklin Lakes, NJ, USA) was added, and a further 15 min incubation in the dark was performed. Samples were analyzed in a flow cytometer immediately after lysis. A FACS Canto II flow cytometer (BD Biosciences, Franklin Lakes, NJ, USA) was used for analysis. The FACSDiva software (version 8.0.1) was used for analysis of CRegPs expression in CD4^+^ and CD8^+^ T cell and B cell populations. The gating strategy is shown in Supplemental Figure S1.

### 2.3. C3a and C5b-9 Measurement

For the assessment of C3a and C5b-9 levels, blood samples were processed as previously described [17]. Specifically, whole blood samples were collected in EDTA-coated tubes and stored at room temperature until further processing. All samples were processed within approximately one hour of collection. Blood samples were centrifuged for 15 min at 1000× *g* at 4 °C. Plasma samples were isolated and stored immediately at −80 °C until further use. Samples were thawed on ice. The levels of C3a and C5a were measured by standard enzyme-linked immunosorbent assay (ELISA) methodology. C3a and C5a ELISA kits were obtained from Quidel (San Diego, CA, USA) and measurements were performed according to the manufacturer’s instructions.

### 2.4. Statistical Analysis

Results are reported as absolute numbers, mean and standard deviation, or median and interquartile ranges, as appropriate. Statistical analysis was performed using GraphPad Prism 9.0 software for Windows. The data were tested for normality using the Shapiro-Wilks test. Outliers were identified and excluded from the analysis. An unpaired *t*-test or Mann-Whitney was used in the case of data displaying normality or not, respectively. One-way ANOVA was used in cases of more than two group comparisons. A non-parametric Spearman correlation was computed as values in the specific groups did not show a normal distribution. A *p* value *p* < 0.05 was considered statistically significant.

## 3. Results

### 3.1. Clinical Characteristics of Patients

A total of 20 patients were recruited in the study, eight of whom were (40%) female and 12 (60%) were male. The clinical data of the patients before EPO and after EPO therapy are shown in Table 2. 

All patients had CKDs of various pathologies in stages 3b to 5, as shown in Table 1. As mentioned previously, the various pathologies included nephrosclerosis, diabetes, polycystic kidney disease, membranous nephropathy, and IgA nephropathy and affected equally female and male patients of the study, with the exception of polycystic kidney disease, which was the main cause of CKD in two male patients. All patients were categorized by the MDRD equation according to KDIGO guidelines [16]. Differences between patients who had similar levels of creatinine but higher or lower levels of Hb than expected are due to individuality and the fact that EPO is not exclusively produced by the kidneys [18,19].

Hematocrit and hemoglobin levels were significantly decreased in patients compared to the control group prior to the initiation of therapy (Supplemental Figure S2). Following the erythropoietin course of treatment, hematocrit and hemoglobin levels increased significantly but remained significantly reduced compared to the healthy controls (Supplemental Figure S2).

Four patients took the longest time to reach Hb goals. Three of these patients suffered from β-thalassemia minor, whereas the fourth suffered from latent myelodysplastic syndrome, and our observations are supported by similar findings in the literature [20,21]. We also observed that patients with lower eGFR values required a longer duration and EPO dose in order to achieve the goal of 11–11.5 g/dL hemoglobin levels.

### 3.2. Effect of EPO on Complement in CKD Patients

**Figure 1:**
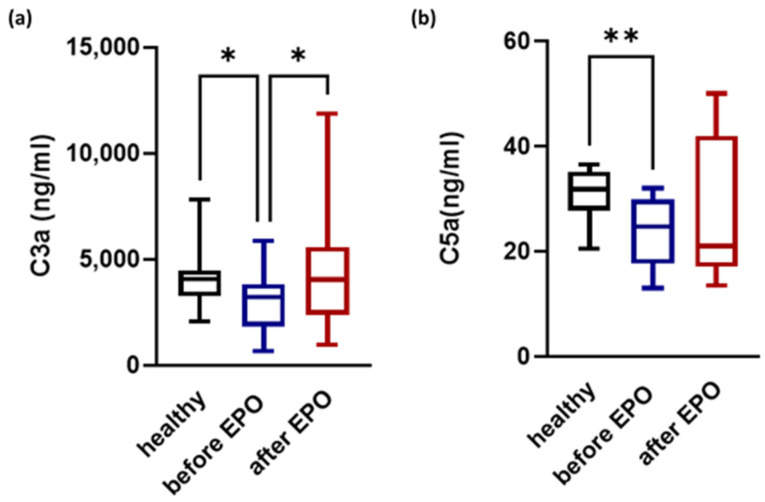
Increased levels of C3a and C5a in CKD patients in response to erythropoietin. (**a**) C3a and (**b**) C5a levels were measured prior to and following the completion of EPO therapy and compared to the healthy controls. Statistical analysis was performed by one-way ANOVA. Post-hoc analysis was carried out with an uncorrected Fisher’s test. * *p* < 0.05; ** *p* < 0.01.

C3a and C5a levels were measured at both time points (prior to and at the completion of the erythropoietin course of therapy). Reduced levels of C3a and C5a were observed in CKD patients prior to the initiation of EPO in comparison to healthy controls (Figure 1a and Figure 1b, respectively). C3a and C5a levels increased in response to EPO and returned to levels similar to those of healthy controls. A statistically significant increase was observed for C3a levels in patients following the completion of EPO therapy compared with the initial time point. 

**Figure 2:**
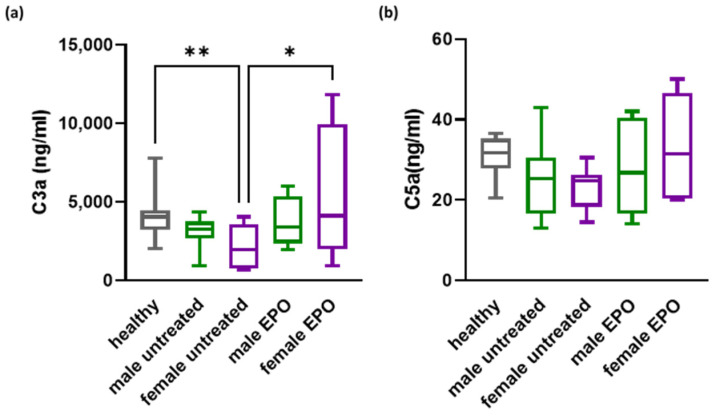
Effect of EPO on C3a and C5a levels in female and male CKD patients. (**a**) C3a and (**b**) C5a levels in female and male CKD patients following EPO administration. Statistical analysis was performed by one-way ANOVA. Post-hoc analysis with uncorrected Fisher’s test. * *p* < 0.05; ** *p* < 0.01.

We next performed a subsequent analysis following the subgrouping of patients according to sex. Previous studies have reported differences in various complement factor levels between female and male patients [22,23]. As shown in Figure 2, a statistically significant increase was observed in C3a levels in female patients upon completion of EPO therapy. 

Although an increase in C5a levels in response to EPO was observed for both female and male patients, this did not reach statistical significance, and equal numbers of patients in the male and female groups showed responses to EPO, indicating that gender does not affect the C5a response to EPO.

**Figure 3:**
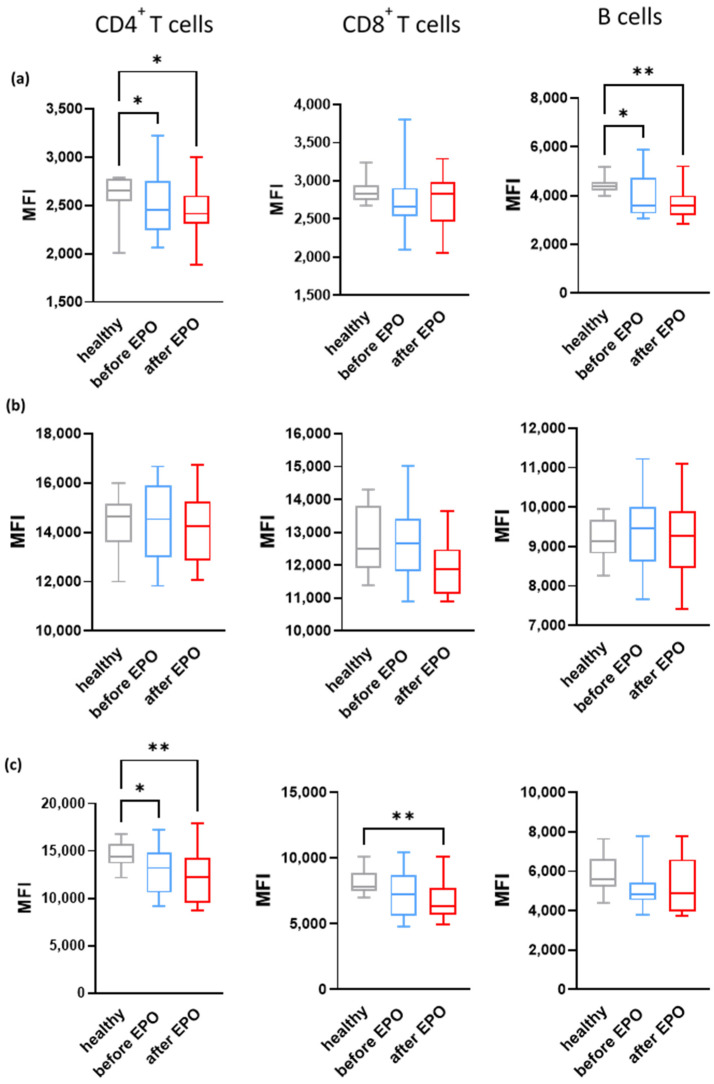
Effect of erythropoietin on complement regulatory protein expression on CD4^+^, CD8^+^ T cells, and B cells in CKD patients. (**a**) CD55, (**b**) CD46, and (**c**) CD59 levels were determined on CD4^+^, CD8^+^ T cells, and B cells by flow cytometric analysis. Results are represented as mean fluorescence intensity (MFI). Statistical analysis was performed with one-way ANOVA. Post-hoc analysis with an uncorrected Fisher’s test. * *p* < 0.05; ** *p* < 0.01.

We next investigated whether EPO therapy exerts an effect on CRegPs expression. In order to achieve that, FACS analysis was performed for the assessment of the expression levels of CD55, CD46, and CD59 on T and B lymphocytes before and after EPO therapy. A trend of reduced expression of CregPs was observed for CD55 in CD4^+^ and CD8^+^ T cells, as well as in B cells (Figure 3a). A statistically significant decrease in CD55 was shown in CD4^+^ T cells both before and after EPO therapy compared with healthy controls. The same was observed in the B cell population (Figure 3a). CD46 levels remained largely unaffected by EPO in all cell populations examined (Figure 3b) and were fairly similar to those of healthy controls, with the exception of CD8^+^ T cells, in which a trend of reduced CD46 levels was observed following EPO therapy, albeit, non-significant (Figure 3b).

A trend of reduced expression was also observed for CD59 in all cell populations, with a statistically significant reduction in CD4^+^ and CD8^+^ T cells of patients at the completion of EPO therapy compared with healthy controls (Figure 3c). 

### 3.3. Correlations of Complement Factors and Regulatory Proteins with Clinical Characteristics

**Figure 4:**
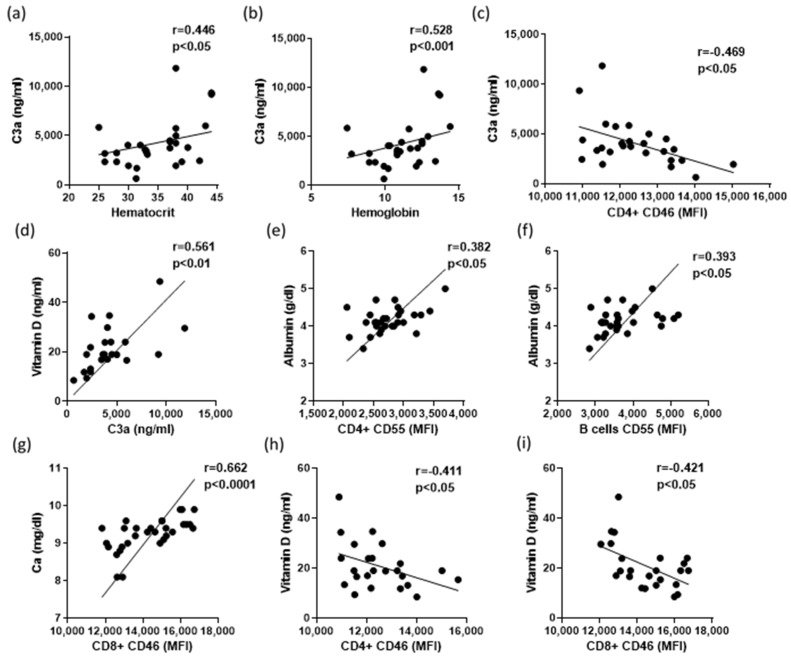
Complement factors and regulatory protein correlations with patient clinical parameters following EPO therapy. Spearman’s correlation of (**a**) C3a and hematocrit levels (**b**) C3a and hemoglobin levels (**c**) C3a levels and CD46 expression on CD4^+^ T cells, (**d**) C3a and Vitamin D levels, (**e**) Albumin and CD55 expression on CD4^+^ T cells, (**f**) Albumin and CD55 expression on B cells, (**g**) Ca levels and CD46 expression on CD8^+^ T cells, (**h**) Vitamin D levels and CD46 expression on CD4^+^ T cells and (**i**) Vitamin D levels and CD8^+^ T cells. All correlations were performed with Spearman’s correlation.

We next assessed whether the changes observed in complement factors and regulatory protein levels could be associated with the patients characteristics. As shown in Figure 4, a positive correlation was observed for C3a with both hematocrit and hemoglobin (r = 0.446; *p* < 0.05 and r = 0.528; *p* < 0.001, respectively) as well as with vitamin D (r = 0.561; *p* < 0.01). A negative correlation was observed for C3a levels with CD46 expression in CD4^+^ T cells (r = −0.469; *p* < 0.05).

CD55 expression in CD4^+^ T cells and B cells correlated positively with albumin (r = 0.382; *p* < 0.05 and r = 0.393; *p* < 0.05, respectively), while a positive association with Ca was observed for CD46 expression in CD8^+^ T cells (r = 0.662; *p* < 0.0001). Finally, CD46 in CD4^+^ and CD8^+^ T cells negatively correlated with the vitamin D levels of CKD patients (r = −0.411; *p* < 0.05 and r = −0.421; *p* < 0.05, respectively) (Figure 4). 

## 4. Discussion

The present study aimed to identify the effect of erythropoietin on the complement system in patients with chronic kidney disease. EPO, primarily produced in the kidneys, regulates red blood cell production and oxygen transportation. However, its functions extend beyond hematopoiesis, encompassing tissue protection, anti-inflammatory properties, and immune modulation. In CKD, EPO deficiency is common, contributing to anemia and exacerbating disease progression [24]. Anemia-induced hypoxia within the kidneys contributes to renal tissue damage and fibrosis, exacerbating CKD progression. Hypoxia triggers a cascade of events leading to increased production of pro-fibrotic factors, such as transforming growth factor-beta (TGF-β) and connective tissue growth factor (CTGF), promoting the accumulation of extracellular matrix proteins and renal fibrosis [25].

Additionally, anemia-induced hypoxia disrupts endothelial function within the renal vasculature, leading to endothelial dysfunction and impaired vascular homeostasis. Endothelial dysfunction is characterized by increased production of vasoconstrictors, such as endothelin-1, and decreased production of vasodilators, such as nitric oxide (NO), resulting in vasoconstriction and reduced renal blood flow. Reduced renal perfusion exacerbates renal hypoxia and contributes to tissue damage and fibrosis, accelerating CKD progression [26].

The dysregulation of both EPO production and complement system activation is implicated in various diseases, including CKD, in which inflammation and tissue damage are prominent features. Previous studies have focused on the effect of erythropoietin on CregPs expression on erythrocytes. Ohi et al. investigated the levels of CD55, CD59, and CR1 on erythrocytes and revealed an increase in all CregPs expressions in response to erythropoietin [12] and a decrease in cases of therapy discontinuation. The direct effect of EPO on erythrocyte CregPs expression was implicated as a mechanism contributing to the overall efficacy of EPO therapy in CKD patients [12]. However, the effect of EPO on other blood cell types was not assessed. Another study investigated the levels of CregPs in various blood cell populations in CKD patients [13]. The study identified a slight decrease in CD35 and CD59 in adult CKD patients compared to the healthy controls; however, this study did not investigate the changes in complement factors or the link with CRegPs changes [13]. 

Our study is the first, to our knowledge, to investigate the effect of EPO on complement in CKD patients by assessing both levels of complement factors and levels of CRegP expression. We identified an increase in C3a and C5a levels in plasma samples of patients upon the completion of EPO with a concurrent decrease in CD55 and CD59 expression in CD4^+^, CD8^+^ T cells, and B cells. Our observation suggests that the decrease in CD55 and CD59 observed in these cells may contribute to the increase in C3a and C5a levels observed at the same time point. CD46 expression levels were shown to be fairly unaffected by EPO and remained stable, with the exception of CD8^+^ T cells, in which a small decrease was observed. However, this could be due to the relatively small number of patients included in our study, and larger differences might be easier to detect in larger cohorts. 

We also identified a negative correlation of C3a with CD46 in CD4^+^ T cells, but no other correlations could be observed between the two complement factors chosen and the CRegPs on the specific cell types. A strong and statistically significant correlation of CD46 on CD8^+^ T cells was observed with Ca levels (r = 0.393; *p* < 0.0001). It has previously been reported that CD46 is a regulator of lymphocyte activation [27]. Furthermore, CD46 has been reported to be involved in Ca efflux [28]. Therefore, the correlation observed in our study might indicate a potential association of CD46 with mediating Ca efflux on CD8^+^ T cells in response to EPO therapy. Interestingly, C3a was also found to correlate with vitamin D with a simultaneous inverse relationship of vitamin D, with CD46 on both CD4^+^ and CD8^+^ T cells. A cross-talk of CD46 with vitamin D in T cells has previously been reported in multiple sclerosis and COVID-19 [29] raising the question of whether similar link between CD46 and vitamin D in kidney disease could be present. 

Our observation of increased C3a and C5a levels in response to EPO suggests that EPO possibly activates the complement system with a restrictive effect on CRegPs expression in blood cells. Although we did not investigate the effect of EPO in other cell populations, our findings indicate that, following EPO therapy, complement is overactivated. In our study, the levels of complement (C3a and C5a) returned to those of the healthy controls; however, it was obvious that, in certain patients, C5a levels increased much higher than the levels observed in healthy individuals. C5a is an anaphylatoxin with strong chemoattractant properties, such as the recruitment of inflammatory cells, monocytes, T lymphocytes, and eosinophils and the activation of phagocytic cells, thus possibly contributing to inflammation and tissue damage. Furthermore, the increased levels of C5a in these patients could be an indicator of increased terminal component levels (C5b-9) and therefore lysis and tissue damage. However, C5b-9 levels were not directly assessed in our study. A significant increase in C3a levels was also observed in female patients following EPO therapy when patients were sub-grouped according to sex. However, this increase was mainly attributed to two patients with relatively high C3a values, whereas the rest of the patients showed C3a values similar to those of the male patients. Including a larger number of patients would possibly allow us to observe whether female patients fall into separate groups according to the C3a values generated following EPO therapy. A limitation of our study is the small number of patients recruited. However, we have tried to include anemic patients with variable CKD etiology, and our observations hopefully provide useful information on the effect of EPO on complement activation and regulation in these patients. Another limitation concerns the observed significant difference in age between the healthy controls group and the patient group; however, it was very difficult to age match our patients according to our criteria for the healthy controls (no comorbidities and no therapy). Furthermore, we had to omit two patients from the analysis as one of them passed away during the study and the other could not reach our clinic due to COVID-19 restrictions, thus further limiting the power of our analysis. Further research, possibly with additional centers and extended clinical parameter analysis, would probably provide more insight, and allow for the examination of our original observations and the identification of more underlying factors contributing to the effect of EPO on complement cascade changes in these patients. 

## 5. Conclusions

EPO directly affects the complement system in CKD patients. The increase in complement factor levels and a simultaneous decrease in CRegPs in specific blood cell types observed following EPO administration in the present study indicate a potential activation of the complement system in CKD patients in response to EPO. Further research is needed to unravel the underlying mechanisms of EPO-mediated complement activation in these patients. 

## Figures and Tables

**Table 1:** CKD stages and etiology in the study patients.

Cause of CKD	Patients	Percentage (%)	Male	Percentage (%)	Female	Percentage (%)
Hypertensive nephropathy	10	55.55%	4	40%	6	60%
Diabetes	4	22.22%	3	75%	1	25%
Polycystic kidney disease (PKD)	2	11.11%	2	100%	0	0%
Glomerulopathies	2	11.11%	1	50%	1	50%
**CKD Stage**						
3b	7	38.88%	3	42.85%	4	57.15%
4	5	27.77%	2	40%	3	60%
5	6	33.33%	5	83.3%	1	16.66%

**Table 2:** Clinical characteristics of patients before and after EPO therapy.

	Before EPO Treatment	After EPO Treatment	*p* Value
Laboratory Baseline			
**Ht (%)**	30.32 ± 2.641	39.33 ± 2.59	<0.0001
**Hb (g/dL)**	9.82 ± 1.05	12.55 ± 0.9122	<0.0001
**Ferritin (ng/mL)**	125.5 (30.15–189.5)	76.5 (44.25–136)	0.391
**Urea (mg/dL)**	96.08 ± 41.61	101.6 ± 42.5	0.6627
**Creatinine (mg/dL)**	3.146 ± 2.547	3.302 ± 2.628	0.1061
**Albumin (g/dL)**	4.169 ± 0.361	4.133 ± 0.297	0.8402
**eGFR-MDRD (mL/min/1.73 m^2^)**	26.29 ± 13.12	25.83 ± 13.74	0.3316
**PTH (pg/mL)**	104 (67.53–188.8)	140.5 (67.50–236.5)	0.4212
**Ca (mg/dL)**	9.2 ± 0.4147	9.272 ± 0.4336	0.4355
**P (mg/dL)**	4.033 ± 0.8702	4.356 ± 1.030	0.0171
**25(OH)Vitamin.D (ng/mL)**	16.87 ± 6.323	22.03 ± 10.24	0.2324

Data are presented as mean ± SD in the case of normally distributed data or median and interquartile ranges in case of data without normal distribution.

## Data Availability

All data are available in the article.

## Supplementary Materials

The following supporting information can be downloaded at: https://www.mdpi.com/article/10.3390/biomedicines12081746/s1, Figure S1: Gating strategy for CD55, CD46 and CD59 detection in CD4+, CD8+ and B cells by Flow cytometric analysis.; Figure S2: Hematocrit and hemoglobin levels in patients in response to erythropoietin Table S1: List of antibodies utilized for detection of CD55, CD46 and CD59 on CD4+ and CD8+ T cells and B cells by flow cytometric analysis.
