# Supplementary material for: Erythropoietin Effect on Complement Activation in Chronic Kidney Disease
Source: Biomedicines. 2024 Aug 2;12(8):1746. doi: 10.3390/biomedicines12081746 (PMC11351309; doi:10.3390/biomedicines12081746)
Supplement: Supplementary file 1 [file biomedicines-12-01746-s001.zip › biomedicines-3053505-supplementary.pptx]

## Slide 1
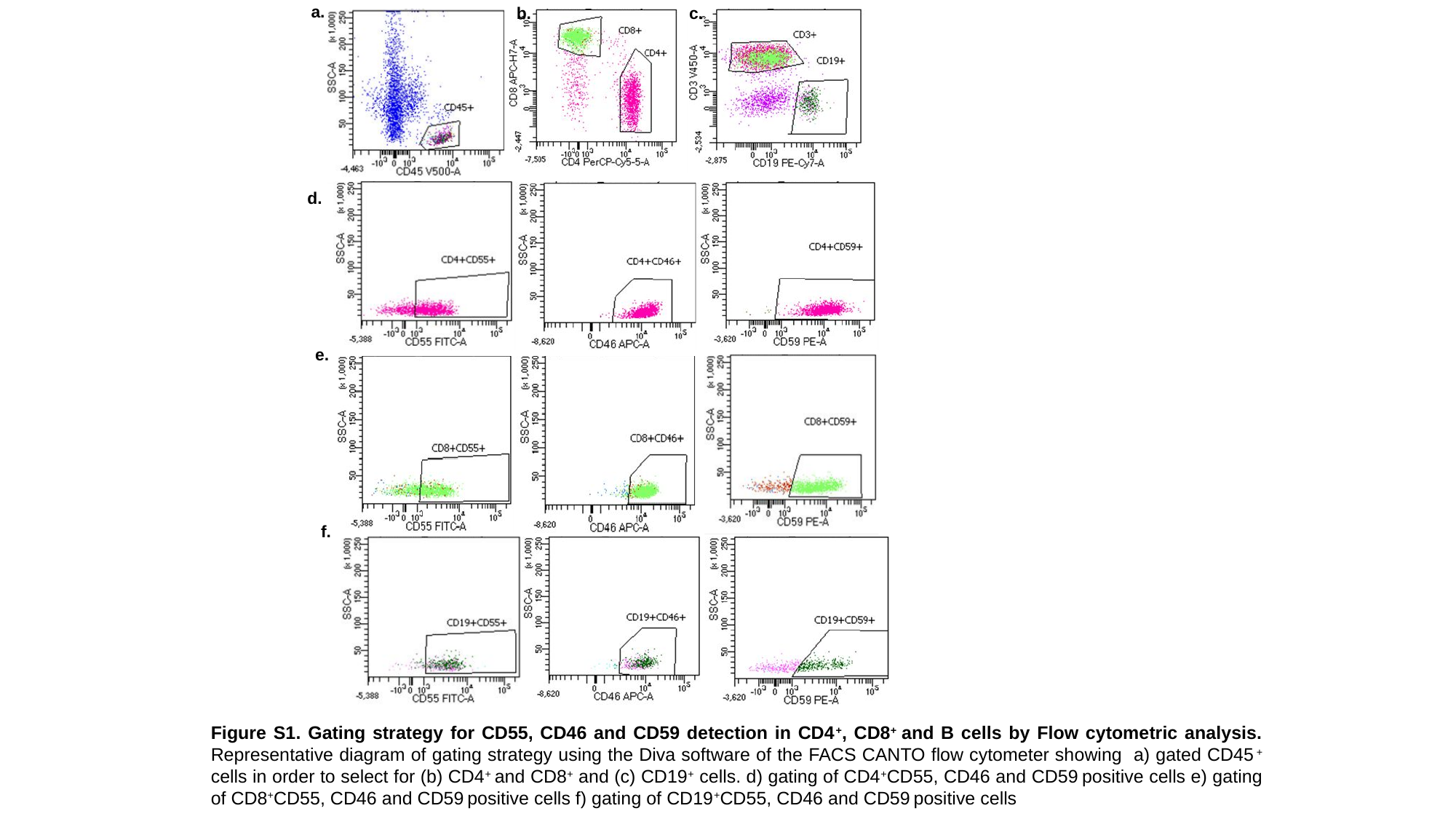

a.
b.
c.
d.
e.
f.
Figure S1. Gating strategy for CD55, CD46 and CD59 detection in CD4+, CD8+ and B cells by Flow cytometric analysis. Representative diagram of gating strategy using the Diva software of the FACS CANTO flow cytometer showing a) gated CD45+ cells in order to select for (b) CD4+ and CD8+ and (c) CD19+ cells. d) gating of CD4+CD55, CD46 and CD59 positive cells e) gating of CD8+CD55, CD46 and CD59 positive cells f) gating of CD19+CD55, CD46 and CD59 positive cells

## Slide 2
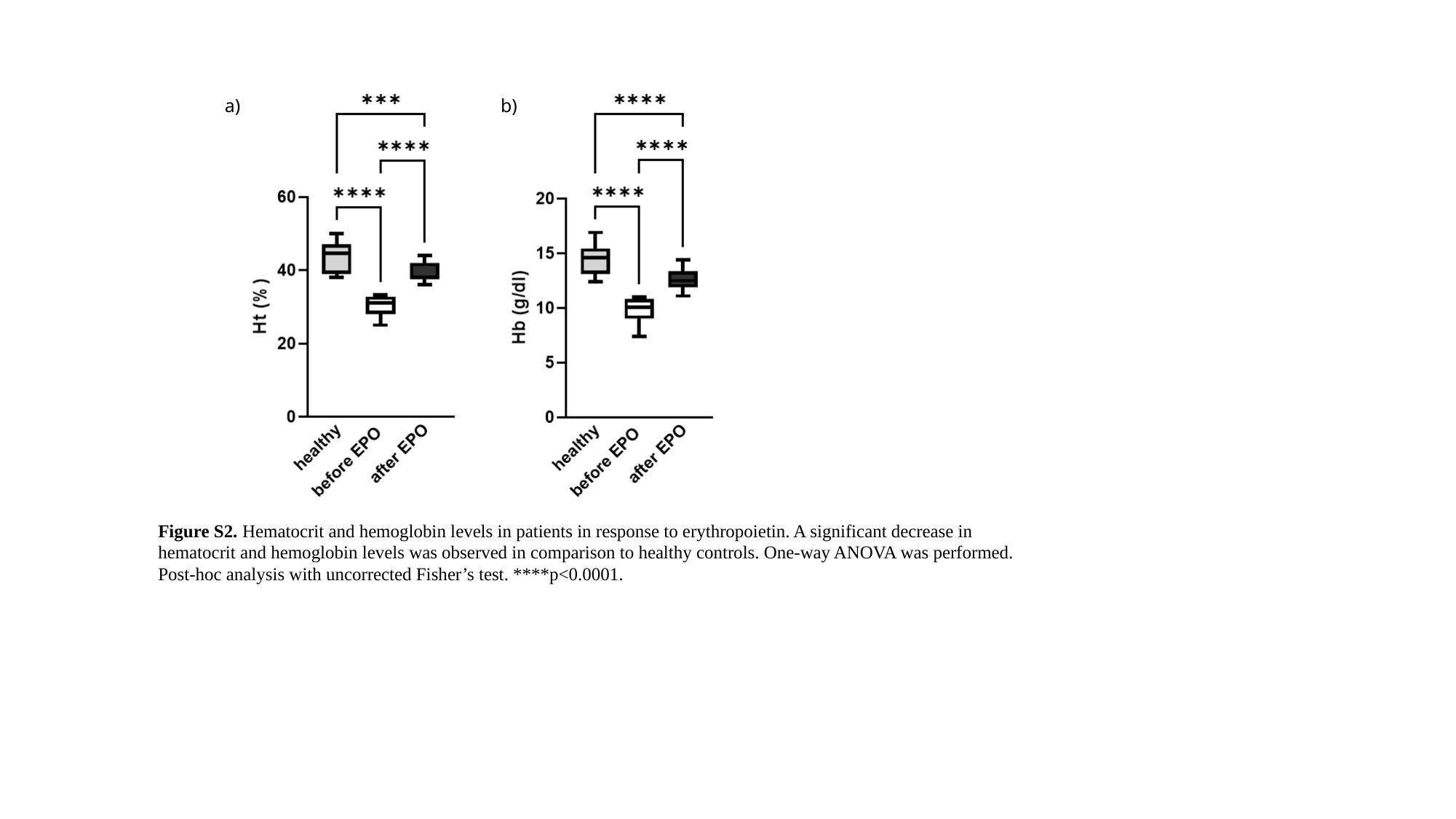

a)
b)
Figure S2. Hematocrit and hemoglobin levels in patients in response to erythropoietin. A significant decrease in hematocrit and hemoglobin levels was observed in comparison to healthy controls. One-way ANOVA was performed. Post-hoc analysis with uncorrected Fisher’s test. ****p<0.0001.

## Slide 3
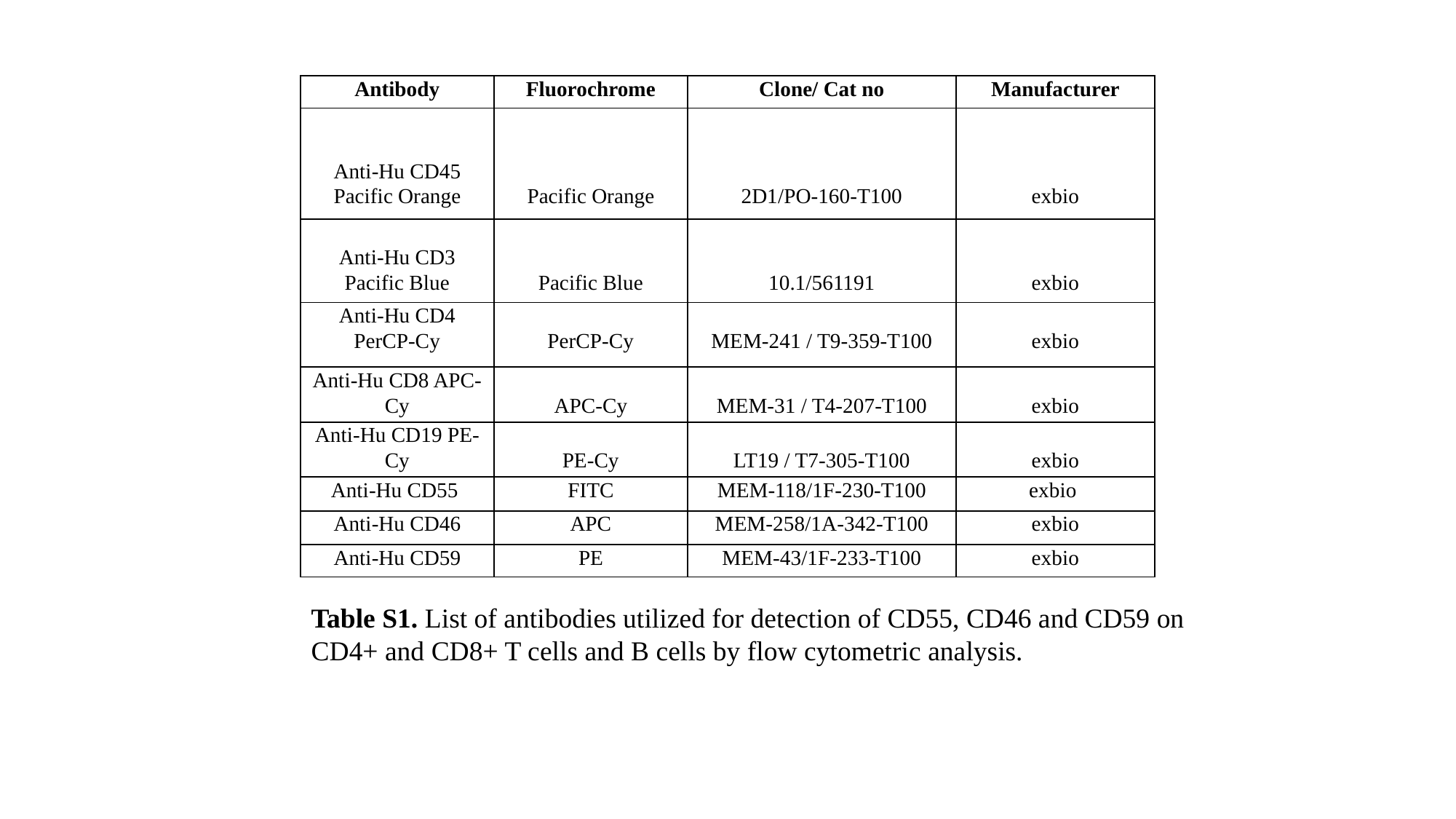

| Antibody | Fluorochrome | Clone/ Cat no | Manufacturer |
| --- | --- | --- | --- |
| Anti-Hu CD45 Pacific Orange | Pacific Orange | 2D1/PO-160-T100 | exbio |
| Anti-Hu CD3 Pacific Blue | Pacific Blue | 10.1/561191 | exbio |
| Anti-Hu CD4 PerCP-Cy | PerCP-Cy | MEM-241 / T9-359-T100 | exbio |
| Anti-Hu CD8 APC-Cy | APC-Cy | MEM-31 / T4-207-T100 | exbio |
| Anti-Hu CD19 PE-Cy | PE-Cy | LT19 / T7-305-T100 | exbio |
| Anti-Hu CD55 | FITC | MEM-118/1F-230-T100 | exbio |
| Anti-Hu CD46 | APC | MEM-258/1A-342-T100 | exbio |
| Anti-Hu CD59 | PE | MEM-43/1F-233-T100 | exbio |
Table S1. List of antibodies utilized for detection of CD55, CD46 and CD59 on CD4+ and CD8+ T cells and B cells by flow cytometric analysis.
